# Assessing *in vitro* stem-cell function and tracking engraftment of stem cells in ischaemic hearts by using novel iRFP gene labelling

**DOI:** 10.1111/jcmm.12321

**Published:** 2014-06-09

**Authors:** Yingjie Wang, Mi Zhou, Xiaolong Wang, Gangjian Qin, Neal L Weintraub, Yaoliang Tang

**Affiliations:** a Internal Medicine of Traditional Chinese Medicine, Shuguang Hospital of Shanghai University of Traditional Chinese MedicineShanghai, China; b Department of Medicine, University of CincinnatiCincinnati, OH, USA; c Department of Cardiac Surgery, Rui Jin Hospital, School of Medicine, Shanghai Jiao Tong UniversityShanghai, China; d Feinberg Cardiovascular Research Institute, Northwestern University Feinberg School of MedicineChicago, IL, USA; e Vascular Biology Center, Department of Medicine, Medical College of Georgia, Georgia Regents UniversityAugusta, GA, USA

**Keywords:** stem cells, myocardial infarction, cell transplantation, iRFP, GFP

## Abstract

Near-infrared fluorescence (NIRF) imaging by using infrared fluorescent protein (iRFP) gene labelling is a novel technology with potential value for *in vivo* applications. In this study, we expressed iRFP in mouse cardiac progenitor cells (CPC) by lentiviral vector and demonstrated that the iRFP-labelled CPC (CPC^iRFP^) can be detected by flow cytometry and fluorescent microscopy. We observed a linear correlation *in vitro* between cell numbers and infrared signal intensity by using the multiSpectral imaging system. CPC^iRFP^ injected into the non-ischaemic mouse hindlimb were also readily detected by whole-animal NIRF imaging. We then compared iRFP against green fluorescent protein (GFP) for tracking survival of engrafted CPC in mouse ischaemic heart tissue. GFP-labelled CPC (CPC^GFP^) or CPC labelled with both iRFP and GFP (CPC^iRFP^ ^GFP^) were injected intramyocardially into mouse hearts after infarction. Three days after cell transplantation, a strong NIRF signal was detected in hearts into which CPC^iRFP^ ^GFP^, but not CPC^GFP^, were transplanted. Furthermore, iRFP fluorescence from engrafted CPC^iRFP^ ^GFP^ was detected in tissue sections by confocal microscopy. In conclusion, the iRFP-labelling system provides a valuable molecular imaging tool to track the fate of transplanted progenitor cells *in vivo*.

## Introduction

Cell-based therapy is a promising therapeutic strategy for regeneration of ischaemic and injured tissues. One of the most important challenges for the improvement of cell therapy is to image engrafted cell survival and biodistribution in real time [1]. In mammals, the application of conventional GFP-like fluorescent proteins, including eGFP, DsRed and mCherry, is hampered by the limited depth penetration of visible light in the body [2]. Proteins with excitation and emission maxima within a near-infrared window from ∼650 to 900 nm in which tissues have lower absorbance and less light scattering are more suitable for *in vivo* imaging [3,4]. Recently, a near-infrared fluorescent mutant of the DrBphP bacteriophytochrome from Deinococcus radiodurans, named IFP1.4, was reported to be useful for *in vivo* imaging of adenovirus-infected liver [5]. However, the imaging protocol was complicated by the requirement to inject biliverdin intravenously within 1 hr of imaging IFP1.4. Infrared fluorescent protein (iRFP), which has been used recently to image tumour growth *in vivo*, does not require biliverdin for activation, and iRFP can brightly labels live mammalian cells, with emission and excitation spectra at near-infrared wavelengths that undergo substantially less scattering and absorption than visible light in most tissues [6,7]. These characteristics suggest that iRFP can overcome the limitations of GFP-like fluorescent proteins for imaging engrafted stem cells *in vivo*. In this study, we labelled cardiac progenitor cells (CPC) by using a lentiviral-iRFP system, which enabled us to track labelled cells repeatedly until their death [8]. We found that the iRFP-labelled CPC can be readily detected in the infarcted hearts non-invasively *in vivo* and corresponded with their detection in heart tissue sections by iRFP fluorescence imaging. Lentiviral-iRFP cell labelling technology thus represents a novel approach for monitoring stem-cell homing and survival in living animals, and cell differentiation/apoptosis *ex vivo* by histological staining. These findings broaden the application of iRFP systems for stem-cell studies.

## Materials and methods

### CPC culture

Mouse CPC were isolated as previously described [9–11] by using a protocol approved by the Institutional Animal Care and Use Committee of the University of Cincinnati and Georgia Regents University. Briefly, adult mouse hearts were minced to 1 mm^3^ in size and plated on laminin-coated dishes for 2 weeks. The round, phase-bright migrating cells were harvested and filtered with 40 μm cell strainers to avoid heart tissue contamination. Cells were cultured in poly-d-lysine-coated dishes for an additional 2 weeks until cardiospheres formed, which were collected and cultured in fibronectin-coated dishes. Lin^−^ cells were isolated from the cardiospheres through the use of a haematopoietic Lin-depletion cocktail (StemCell Technologies, Vancouver, BC, Canada) according to the manufacturer's protocol. The selected CPC cells were cultured and maintained in complete media containing DMEM/F12, 10% foetal calf serum, 1× L-glutamine-200 mM (100×), 1× 2-mercaptoethanol (Gibco® 55 mM, 1000×) and 1× MEM non-essential amino acids (Gibco®100x, Invitrogen Corporation, Carlsbad, CA, USA).

### Lenti-iRFP vector construction and infection of CPC

The pSin-EF2-iRFP-Puro plasmid was created by amplifying a 963 bp fragment of iRFP cDNA from piRFP (Addgene 31857) into EcoR1 and Spe1 in pSin-EF2-Lin28-Pur (Addgene 16580, 5′ PCR primer: CTAGCAATTGGCCACCATGGCTGAAGGATCCGTCGC; 3′ PCR primer: CTAGACTAGTTCACTCTTCCATCACGCCGA). Viral vectors encoding GFP and iRFP were produced by transfection of 293FT cells with the lentiviral backbone plasmid pRRLSin.cPPT. PGK-GFP. WPRE (Addgene 12252) or pSin-EF2-iRFP-Puro, an envelope plasmid (pMD2.G), and a packaging plasmid (psPAX2) with Fugene HD (Roche Diagnostics GmbH, Mannheim, Germany). Virus-containing medium was collected 48 hrs after transfection on 2 consecutive days, passed through a 0.45 μm filter to remove cell debris, and concentrated by ultracentrifugation. Lentiviral vectors expressing iRFP or GFP with 8 μg/ml polybrene (Sigma-Aldrich, St. Louis, MO, USA) were applied to mouse CPC cells; At 72 hrs after infection, the medium was replaced.

### Identification of iRFP^+^ CPC by FACS

To characterize the cells that express iRFP from the CPC cultures (CPC^iRFP^), we dissociated them into a single-cell suspension by using trypsin. Prior to sorting, aggregates were removed by passing through a 40 μm cell strainer. Fluorescence-activated cell sorting (FACS) was carried out on BD FACSAria II, gated for near-infrared fluorescence (Alexa 680). Non-infected CPC were used as negative controls. After FACS sorting, CPC^iRFP^ were seeded on culture dishes and examined by fluorescent microscopy (EVOS® Cell Imaging Systems, Life Technologies, Carlsbad, CA, USA) by using a Cy5 filter.

### Cell migration assay

Cell migration assays were performed by using Oris™ Pro Cell Migration Assay kit (Platypus Technologies, LLC, Fitchburg, WI, USA) formatted for 96-well plates. Non-toxic biocompatible Oris stoppers were inserted into each well to form a cell-free zone on cell culture surfaces before adding the CPC^iRFP^. Then, 3 × 10^4^ CPC^iRFP^ suspended in DMEM with 2% FBS were added and incubated for 2 hrs to allow cell attachment. Plate scanning was performed using an Odyssey system (Li-COR Biosciences, Lincoln, NE, USA) to establish a pre-migration reference. The stoppers were then removed and plates transferred to a tissue culture incubator (37°C in 5% CO_2_) to allow cell migration into a central detection zone. After 60 hrs of incubation, images were re-captured.

### *In vivo* NIRF imaging of intramuscular injected CPC

For *in vivo* imaging of engrafted cells, eight different dosages of CPC^iRFP^ in 25 μl DMEM (5 × 10^5^, 2.5 × 10^5^, 1.25 × 10^5^, 6.25 × 10^4^, 3.12 × 10^4^, 1.56 × 10^4^, 7.8 × 10^3^, 3.9 × 10^3^ cells) were intramuscularly injected into both hindlimbs of adult C57Bl/6 mice (JAX Laboratory, Bar Harbor, ME, USA). Imaging was performed at 1 hr after cell transplantation in anesthetized mice by using a Kodak In-Vivo MultiSpectral Imaging System FX (2D; Kodak, Rochester, NY, USA) in epifluorescence mode equipped with 620/20 nm (centre wavelength/full width at half maximum) and 720/20 nm filters for excitation and emission, respectively. Images were taken with 60 sec. exposure. The signal intensity is represented by radiance and encoded by pseudocolours on the iRFP image. All animal experiments were performed in a facility approved by the Association for Assessment and Accreditation of Laboratory Animal Care by using protocols approved by the University of Cincinnati, and Georgia Regents University IACUC Committees.

### Myocardial infarction model, and *in vivo* NIRF imaging of intramyocardial injected CPC

Male C57/BL6 mice were anesthetized with ketamine/xylazine (100 mg/kg/10 mg/kg, i.p.) and mechanically ventilated. Myocardial infarction was induced *via* ligation of the left anterior descending coronary artery 2 mm from the tip of the normally positioned left atrium as we have described previously [9,10,12,13]. A 25 μl solution containing 5 × 10^5^ CPC^GFP^ or CPC^GFP iRFP^ in DMEM was injected intramyocardially immediately after induction of MI. Animals were handled according to approved protocols and animal welfare regulations of the Institutional Animal Care and Use Committee of the University of Cincinnati and the Georgia Regents University. Whole-animal infrared fluorescence imaging was performed 3 days after cell transplantation in mice under general anaesthesia (isoflurane) inhalation by using a Carestream MultiSpectral FX (2D) system. The signal intensity is represented by radiance and encoded by pseudocolours on the iRFP image.

### Histopathology

For cell staining, cells were plated on 8-well chamber slides and analysed by Zeiss 710 Laser Scanning Microscope (Carl Zeiss, Thornwood, NY, USA).

For tissue staining, 3 days after intramyocardial injection of CPC^GFP iRFP^, mouse hearts were harvested, fixed in 4% paraformaldehyde for 2 hrs, and equilibrated in 20% sucrose in PBS overnight at 4°C, hearts were then embedded in OCT compound (Sakura, Tokyo, Japan), snap-frozen in liquid nitrogen-chilled isopentane, and cut into 5-μm sections for evaluation of engrafted cells in heart by Zeiss 710 Laser Scanning Microscope.

## Results

### Identification of iRFP-labelled CPC by FACS and fluorescent microscopy

**Fig. 1:**
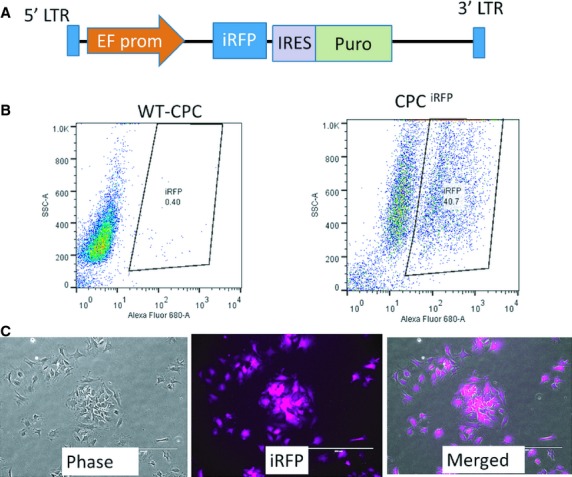
Identification of CPC^iRFP^ by FACS and fluorescent microscopy. (**A**) Schematic of the lenti-iRFP construct; (**B**) FACS analysis (Alexa 680) of non-infected CPC (WT-CPC) and CPC that stably express iRFP following infection with the lenti-iRFP construct (CPC^iRFP^); (**C**) sorted CPC^iRFP^ were imaged by fluorescent microscopy by using a Cy5 filter.

We constructed a lentiviral vector containing the iRFP gene to stably express iRFP protein in mouse CPC (Fig. 1A), and cells successfully infected with lenti-iRFP vector were characterized by FACS. As shown in Figure 1B, about 40% of the CPC exhibited near-infrared range fluorescence, suggesting adequate expression of iRFP protein. To purify the iRFP-expressing CPC, we subjected them to FACS sorting. Following the sorting, 100% of the cells exhibited bright near-infrared fluorescence when fluorescent microscopy (Fig. 1C). Thus, lentiviral-iRFP vector can be used to label CPC, which can be sorted by FACS and imaged by fluorescent microscopy.

### Using iRFP labelling for cell quantification and cell migration assay *in vitro*

**Fig. 2:**
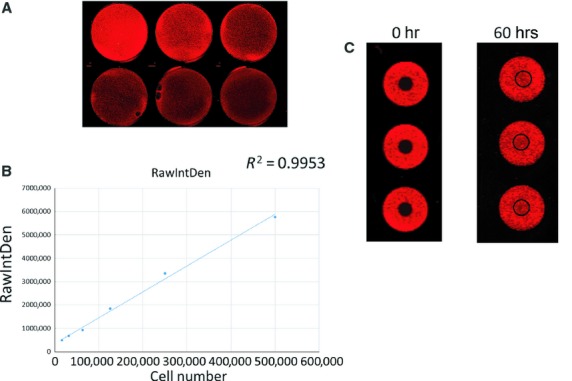
Real-time assessment of cell migration *in vitro* by near-infrared imaging scan. (**A**) CPC^iRFP^ were seeded at six different dilutions onto 6-well plates and subjected to scanning at 700 nm by using an Odyssey Infrared imaging system; (**B**) Assessment of infrared signals showed a robust linear correlation (*R*^2^ = 0.9953) between the number of cells seeded and the near-infrared signal; (**C**) The Oris™ Cell Migration Assay was used to assess cell migration of CPC^iRFP^ by using an Odyssey Infrared imaging system in plate reading format. Note that after 60 hrs migration, the detection zones were covered with CPC^iRFP^.

We first evaluated the correlation between near-infrared signal and the number of seeded cells. Our data showed a robust linear correlation between the number of cells (x) and near-infrared signal values (y), with a square of the correlation coefficient (*R*^2^) of 0.9953 (Fig. 2A and B), suggesting that the iRFP molecular signal enables accurate cell quantification *in vitro*, which is important for assessing cell proliferation.

To test whether iRFP labelling can be used for *in vitro* stem-cell migration assay, we evaluated cell migration by using an Oris Universal Migration Assembly Kit. As demonstrated in Figure 2C, after 60 hrs, CPC^iRFP^ migrated into the detection zone, which can be examined over time by infrared imaging, suggesting that iRFP molecular labelling enables real-time assessment of cell migration *in vitro*.

### *In vivo* near-infrared fluorescent (NIRF) imaging of engrafted CPC

Retention of transplanted stem cells *in vivo* varies depending on the organ into which the cells are transplanted. In the heart, transplanted stem cells can be forced out of the needle track by pressure induced during cardiac systole. Therefore, we examined CPC^iRFP^ in two mouse models: (1) first, we delivered cells to the hindlimb musculature, which is not subjected to the high pressure of the beating heart; (2) second, we intramyocardially injected cells into infarcted hearts to evaluate the efficiency of iRFP labelling on cell tracking in the setting of high intramural pressure.

**Fig. 3:**
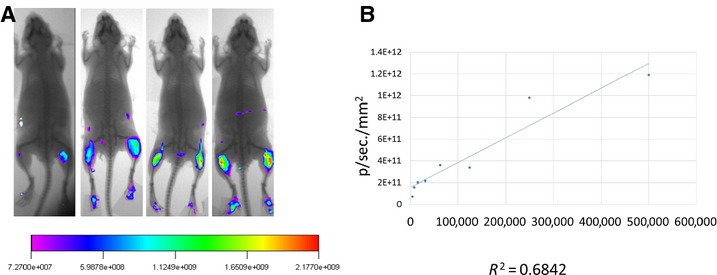
Imaging and quantifying engrafted CPC^iRFP^ in hindlimb muscles. (**A**) CPC^iRFP^ at eight different dilutions were intramuscularly injected into both right and life sides of mouse hindlimb muscles, and whole-animal infrared fluorescence imaging of engrafted CPC^iRFP^ was performed. The fluorescence image was overlaid on an X-ray by using a KODAK In-Vivo Multispectral FX Image 2D Station; (**B**) assessment of infrared fluorescence signals showed a good linear correlation (*R*^2^ = 0.68) between the cell number (*x*-axis) and *in vivo* near-infrared signal (*y*-axis, p/s/mm^2^).

In the hindlimb musculature model, infrared imaging indicated that iRFP fluorescence from CPC can be readily detected after intramuscular injection. Quantitative analysis of infrared signals showed a good linear correlation between NIRF signals (p/sec./mm^2^) and injected cell numbers (*R*^2^ = 0.6842; Fig. 3A and B), suggesting that iRFP cell labelling can be used for quantification of engrafted cell in a low pressure *in vivo* model.

**Fig. 4:**
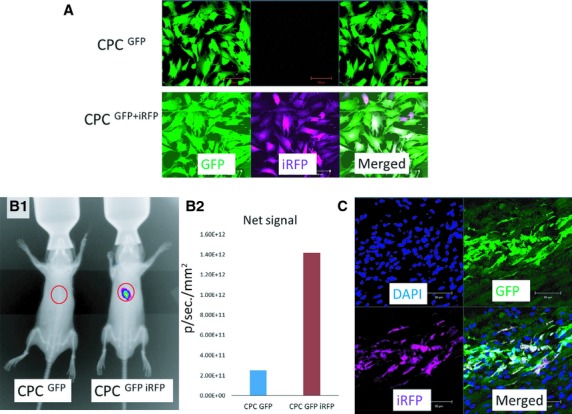
Comparison of iRFP against GFP for tracking engrafted CPC in mouse hearts. (**A**) confocal microscopy of live CPC^GFP^ or CPC^GFP^ ^iRFP^; (**B1**) Near-infrared fluorescence *in vivo* imaging in mice following myocardial infarction and transplantation of CPC^GFP^ or CPC^GFP^ ^iRFP^ intramyocardially into the peri-infarct region. The fluorescence image was overlaid on an X-ray by using a KODAK In-Vivo Multispectral FX Image 2D Station. Three days after cell transplantation, whole-animal near-infrared fluorescence imaging shows that only CPC^GFP^ ^iRFP^ can be detected; (**B2**) quantification of *in vivo* imaging; (**C**) 3 days after cell transplantation, mouse hearts transplanted with CPC^GFP^ ^iRFP^ were analysed by confocal fluorescent microscopy, which confirmed that iRFP has similar efficiency for identification of engrafted CPC compared with GFP.

To determine whether lenti-iRFP cell labelling can be used for *in vivo* tracking of cells in the beating heart after cell transplantation, CPC were genetically modified with GFP alone (CPC^GFP^) or both GFP and iRFP (CPC^GFP^ ^iRFP^) by lentiviral vector (Fig. 4A). Mice were subjected to left anterior descending artery ligation and intramyocardial injection of CPC^GFP^ or CPC^GFP^ ^iRFP^ respectively. We performed non-invasive infrared fluorescence and X-ray image overlays at day 3 after cell transplantation to track the survival of transplanted cells *in vivo*. A strong near-infrared signal was observed in mice treated with CPC^GFP^ ^iRFP^, but no signal was detected in mice treated with CPC^GFP^ (Fig. 4B1-2), suggesting iRFP cell labelling enables the tracking of engrafted CPC non-invasively after cell therapy in mice after myocardial infarction.

### Histological identification of engrafted iRFP-labelled CPC in ischaemic myocardium

To determine whether engrafted cells can be identified in tissue sections by iRFP fluorescence, we harvested hearts transplanted with CPC^GFP iRFP^ 3 days after cell delivery, and analysed the iRFP fluorescence by confocal fluorescence microscopy by using GFP as a reference. As shown in Figure 4C, iRFP signals in explanted heart tissues were co-expressed with GFP signals, suggesting that engrafted CPC can also be detected by their iRFP fluorescence in heart tissues.

## Discussion

Near-infrared-based fluorescence imaging using iRFP gene labelling is a novel technology with potential utility for *in vivo* applications. Here, we found that iRFP-expressing progenitor cells can be detected by FACS at near-infrared fluorescent wavelengths and imaged by fluorescent microscopy by using a Cy5 filter. Also, infrared scanning enabled us to perform real-time assessment of progenitor cell migration *in vitro*. Importantly, iRFP-expressing CPC transplanted to the hindlimb and ischaemic heart were readily detected by whole-animal multispectral imaging. These findings suggest that iRFP fluorescence imaging is a useful approach to track survival of engrafted CPC *in vivo*.

Auto-fluorescence from the tissues often interferes with fluorescent signals emanating from conventional GFP-like fluorescent proteins, including eGFP, DsRed, and mCherry. However, interference is considerably less for probes in the near-infrared channel, such as iRFP [14]. Thus, iRFP labelling can aid in identification of the engrafted cells *via* minimizing the effect of background auto-fluorescence of heart tissues [7]. One advantage of GFP labelling is that cells can be further verified by immunodetection by using GFP antibodies, which are commercially available. Currently, iRFP antibodies are not commercially available, although a flag tag can be fused to the 3′ end of iRFP reporter gene to facilitate detection of engrafted cells by immunostaining with an anti-Flag antibody.

One of the primary advantages of lentiviral gene labelling is that the reporter gene can be passed to the daughter cells so that dilution of the fluorescent signal during cell proliferation/division should be markedly reduced [15]. Ramkisoensing *et al*. [16] recently reported, however, that unintended labelling of cells can occur with lentiviral gene labelling. They reported that secondary transduction was completely abolished when lentiviral-infected cells were added to co-cultured cells 14 days after transduction. As our CPCs had been passaged for more than 14 days prior to cell transplantation, horizontal gene transfer of iRFP from CPC to host cardiomyocytes by lentivirus seems highly unlikely.

In summary, our study demonstrates the feasibility of using iRFP reporter genes to monitor the viability of stem cells after transplantation to the myocardium. iRFP fluorescence labelling combines the advantages of GFP for cell imaging and luciferase for non-invasive whole-animal imaging. The use of near-infrared imaging thus appears to be an ideal approach for pre-clinical imaging of engrafted stem cells in ischaemic myocardium.
